# Postless Tape Augmentation for Posterior Cruciate Ligament Reconstruction

**DOI:** 10.1016/j.eats.2022.12.012

**Published:** 2023-03-27

**Authors:** Matthew J. Hartwell, Daniel B. Goldberg, Samuel G. Moulton, Alan L. Zhang

**Affiliations:** Department of Orthopaedic Surgery, University of California–San Francisco, San Francisco, California, U.S.A.

## Abstract

Many techniques have been described for posterior cruciate ligament (PCL) reconstruction, but residual laxity remains an ongoing challenge. Suture or tape augmentation during ligament reconstruction has become a popular option to prevent graft elongation but comes at the expense of additional costs due to implants for augment fixation, and concern for stress shielding of the graft if the augment and graft are not equally tensioned. We introduce a technique for postless tape augmentation during allograft PCL reconstruction that allows for equal tensioning of graft and augment through the use of a sheath and screw construct without the need for additional implants for augment fixation.

## Introduction

Posterior cruciate ligament (PCL) injuries comprise 1 to 44% of acute knee injuries.^1^ Often, PCL injuries occur in the setting of additional ligament injuries with isolated PCL injuries occurring 40% of the time.^1^ Multiligament knee injuries involving the PCL, as well as isolated grade II or III PCL injuries in young active patients typically undergo surgical management.^1^

Several PCL reconstruction techniques have been described, including single- versus double-bundle reconstruction and tibial inlay versus transtibial reconstruction with interference fixation or all-inside suspensory fixation.^2^^,^^3^ Clinical reports have not demonstrated significant differences in outcomes between these various techniques.^4^^,^^5^ Regardless of technique, residual posterior laxity is a common concern following PCL reconstruction. One study reported that 43-62% of patients had a positive posterior drawer postoperatively.^6^

Suture or tape augmentation provides supplemental strength to the graft construct and may improve laxity. Biomechanical studies have reported less graft elongation and greater ultimate strength with suture or tape augmentation for PCL reconstruction.^7^ Current techniques for augmentation describe use of a distal post for suture or tape fixation.^7^ The supplemental distal post fixation increases cost and potentially leads to a tension mismatch between the suture tape and graft. The purpose of this study is to describe a technique for postless tape augmentation during PCL reconstruction that allows for equal tensioning of graft and augment without the need for additional implants for augment fixation.

### Surgical Technique

**Fig 1:**
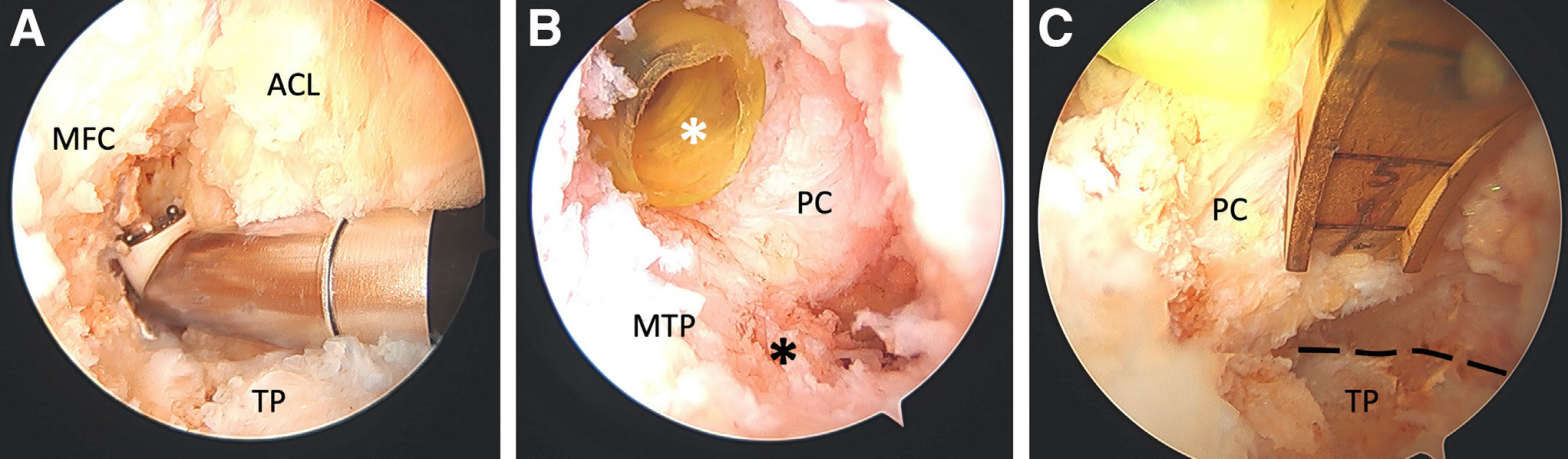
Intraoperative arthroscopic photos of preparing the femoral and tibial footprints for the posterior cruciate ligament (PCL) reconstruction in a left knee. (A) The femoral footprint is prepared with a radio frequency ablation device and shaver to completely clear the femoral wall. (B) The tibial footprint (black asterisk) is prepared with the help of a posteromedial portal (8-mm disposable cannula, white asterisk), which allows for thorough debridement and elevation of the joint capsule from the back wall of the tibia. (C) PCL footprint debridement is complete once the cliff of tibial plateau is visualized (dashed line). ACL, anterior cruciate ligament; MFC, medial femoral condyle; MTP, medial tibial plateau; PC, posterior capsule; TP, tibial plateau.

Video 1 demonstrates our technique for postless tape augmentation during single-bundle allograft PCL reconstruction. The procedure starts by establishing the anterolateral (AL) and anteromedial (AM) arthroscopic knee portals. The anteromedial portal is made below the inferior border of the patella and on the medial border of the patellar tendon. Keeping the AM portal close the patellar tendon allows for improved visualization into the posterior compartment of the knee. Diagnostic arthroscopy is then performed with a 30° arthroscope through the AL portal. Meniscal pathology is first addressed prior to the posterior cruciate ligament reconstruction. We begin the PCL reconstruction by preparing the femoral and tibial footprints. On the femoral side, this includes debriding the PCL remnant with a radiofrequency ablation device and shaver to clear the femoral wall completely (Fig 1A). On the tibial side, this includes establishing a posteromedial (PM) portal that allows for thorough debridement of the PCL stump and elevation of the joint capsule from the back wall of the tibia. First, a Gillquist maneuver is performed with the knee at 90° of flexion from the AL portal to visualize the posteromedial compartment of the knee. Then under direct visualization a spinal needle is placed proximal and posterior to the medial femoral condyle to localize the accessory PM portal. The spinal needle is used to ensure the trajectory to the posterior edge of the tibial plateau is accessible. An 8-mm disposable cannula is then inserted over a switching stick into the accessory PM portal (Fig 1B). A radiofrequency ablation device is used to clear the PCL footprint from the tibial plateau until the cliff of the plateau is reached (Fig 1C).

**Fig 2:**
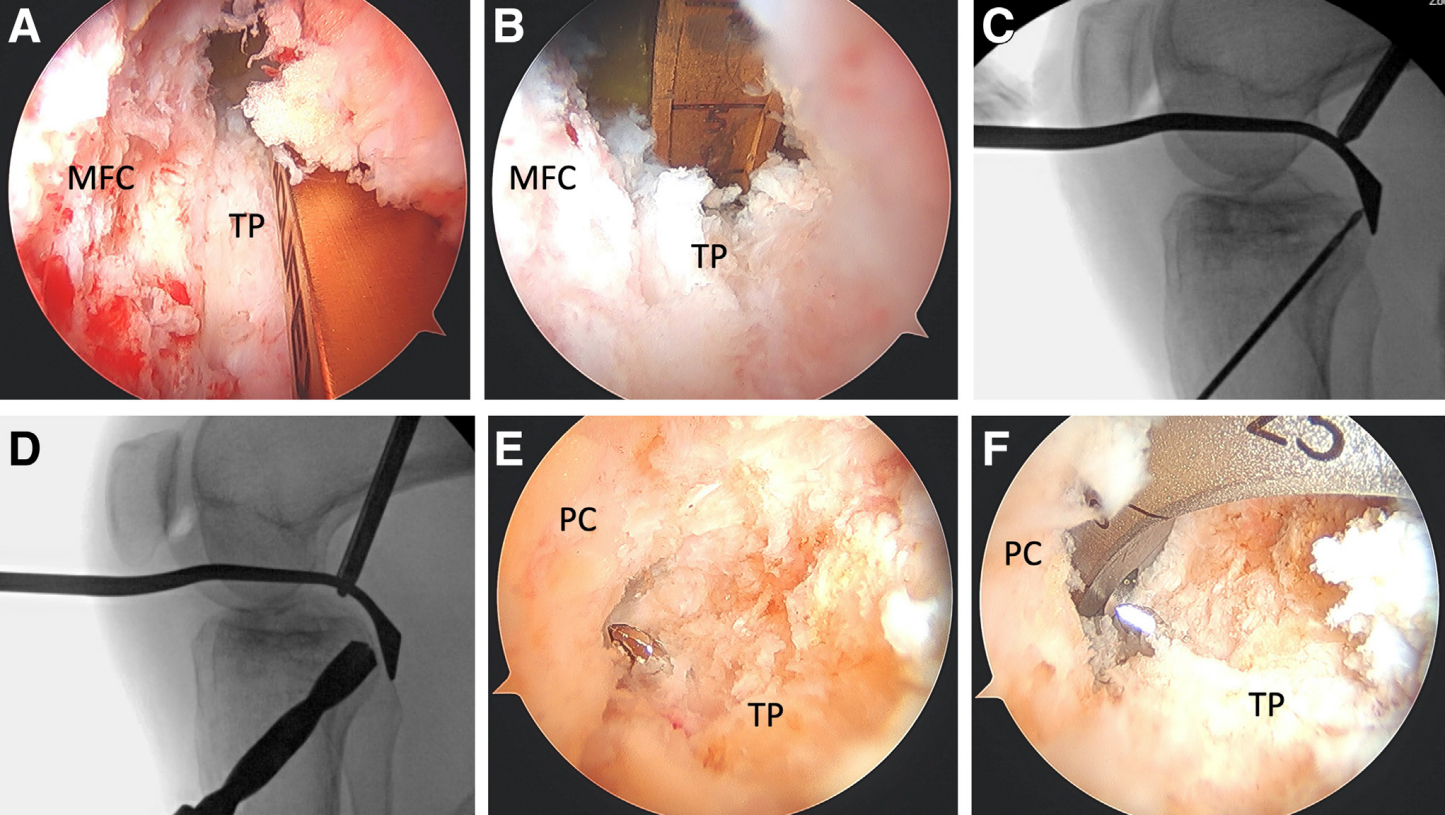
Tibial tunnel preparation begins by placing a posterior cruciate ligament (PCL) guide, set at 50°, through the anteromedial portal (A) and resting it on the PCL footprint (B), which is at the apex of the tibial plateau and close to midline. Correct placement of the aiming arm and pin trajectory are confirmed fluoroscopically. Once the guidewire is in place (C), a PCL elevator with wire catcher is placed over the tip of the guidewire to protect the posterior neurovascular bundle during tunnel drilling (D). Drilling of the guidewire and tunnel reamer should be visualized under arthroscopic visualization (E and F). MFC, medial femoral condyle; PC, posterior capsule; TP, tibial plateau.

We then begin tunnel drilling starting with the tibial side. A PCL drill guide is used, set at 50°, and introduced through the AM portal (Fig 2, A and B). Correct placement of the aiming arm is confirmed fluoroscopically—aimed at the apex of the tibial plateau and close to the midline (Fig 2, C and D). A guidewire is then advanced into the joint under arthroscopic and fluoroscopic visualization, followed by drilling of the tunnel (Fig 2, E and F). A PCL elevator with wire catcher is used to protect the posterior neurovascular bundle, as the reamer is advanced over the guidepin. The tibial tunnel is typically sized to 10 mm, the same size as the bone plug and the Achilles tendon allograft. Once the tunnel has been established, it is plugged, with an instrument such as with a bone tunnel dilator, to prevent fluid extravasation during femoral tunnel preparation.

**Fig 3:**
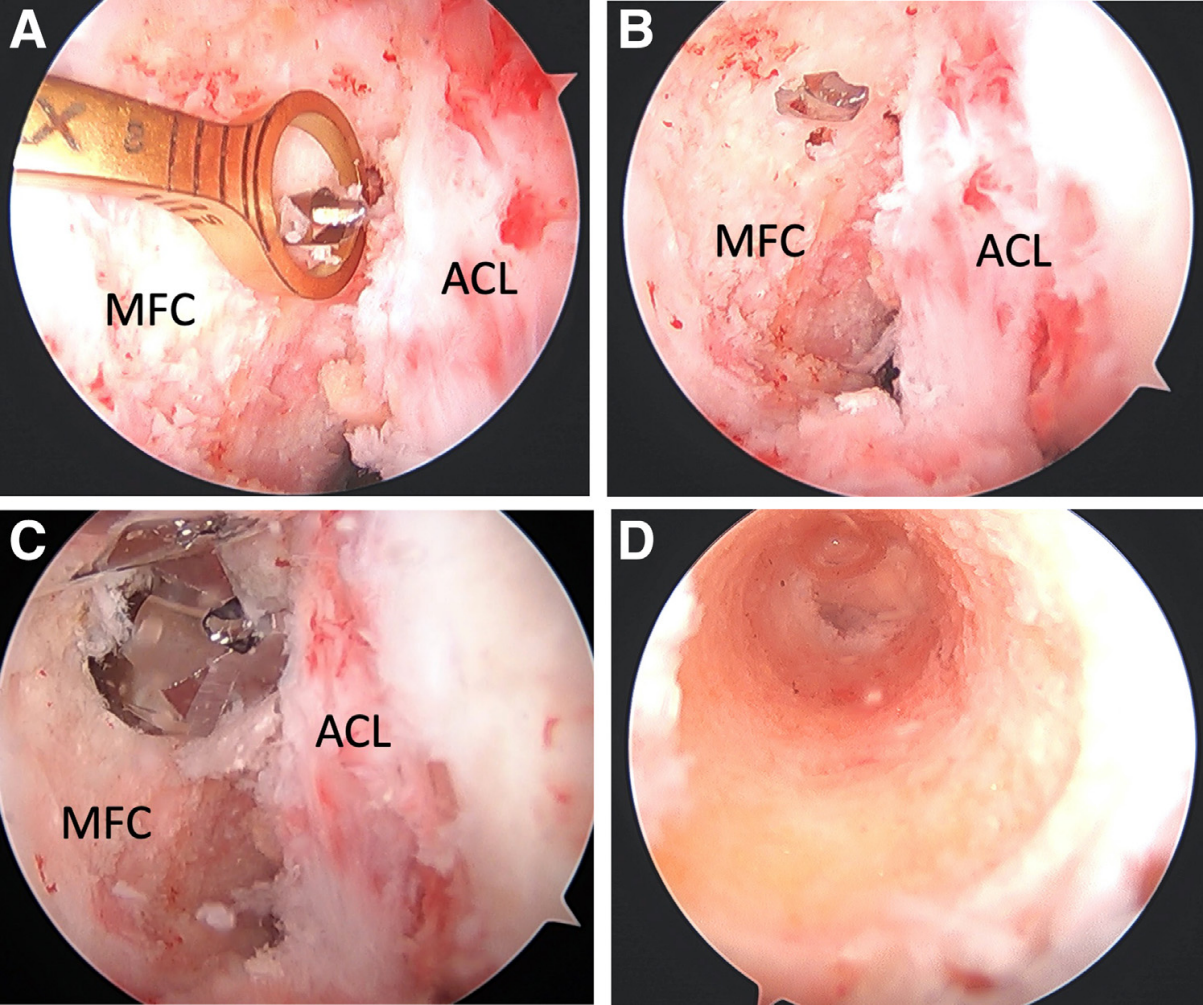
Femoral tunnel drilling begins with (A) placement of a femoral guide, set at 60°, introduced through the anteromedial portal and placed onto the posterior cruciate ligament (PCL) footprint—overlying the anterolateral bundle footprint when performing a single-bundle reconstruction. A 3.5-mm guide pin (B) is drilled first, followed by overdrilling with a 10-mm acorn reamer (C). A clean tunnel can then be visualized arthroscopically (D). ACL, anterior cruciate ligament; MFC, medial femoral condyle.

Femoral tunnel drilling is then performed with a femoral guide, set at 60°, introduced through the AM portal (Fig 3, A-D). Care is taken to ensure that the trajectory of the drill and reamer is posterior and deep to the articular surface of the medial femoral condyle to prevent iatrogenic cartilage injury. The guide is centered within the AL bundle of the PCL for single-bundle reconstruction—approximately 8 mm from the articular cartilage and at the 1 o’clock position. An incision is made over the vastus medialis, and the muscle is split bluntly in line with muscle fibers to expose the bone and avoid bleeding. The tunnel is then drilled from outside-in with a 3.5-mm guide pin, followed by overdrilling with a 10-mm acorn reamer. The acorn reamer is used to chamfer the posterior aspect of the femoral tunnel to aide with graft passage.

**Fig 4:**
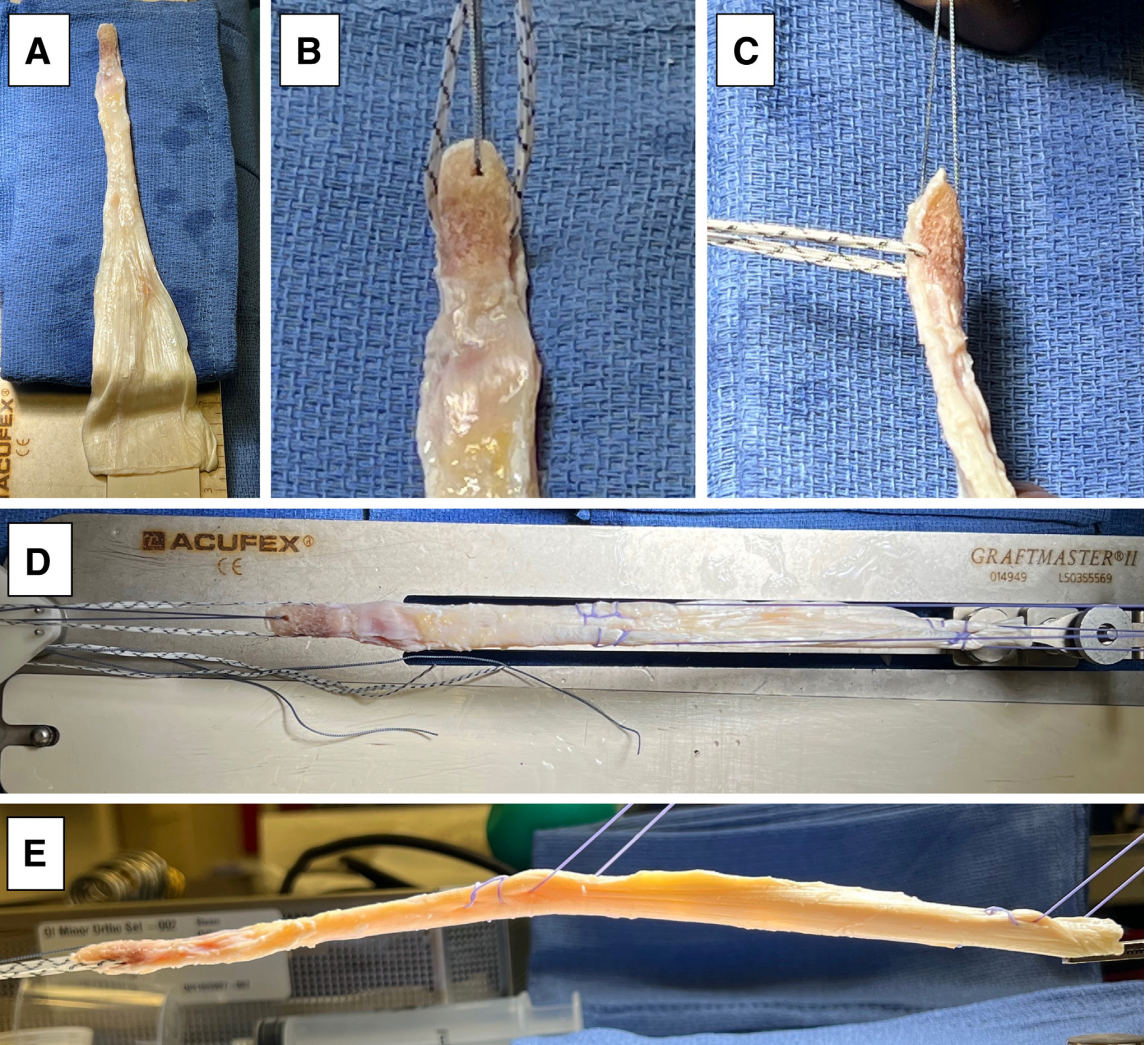
Preparation of an achilles tendon allograft with a bone plug (A). The bone plug portion is prepared by first shaping it to easily pass through a 10-mm tunnel and bulletizing the tip of it to ease passage. A 2.5-mm drill is used to create a hole in the midportion of the bone block in the coronal plane and a 2.5-mm tape suture (Dynatape, Depuy-Mitek, Johnson and Johnson, Raynham, MA) is then passed through the hold in the bone block (B and C). A separate #2 high-tensile strength suture (Orthocord, Depuy-Mitek, Johnson and Johnson) is then passed through the distal end of the bone block (B and C). Two separate #2 high-tensile strength sutures (Orthocord, Depuy-Mitek, Johnson and Johnson) are then passed into the tendinous portion of the graft in a Krackow fashion, with one placed at the end of the graft and the other at the mid-point of the graft (D and E).

An Achilles tendon allograft with a bone plug is then prepared for single-bundle reconstruction with plans for tibial fixation using a metal interference screw and femoral fixation using an interference screw with a sheath. The allograft may be prepared by an assistant concurrently while the surgeon completes the diagnostic arthroscopic and prepares the femoral and tibial tunnels. We begin our graft preparation by starting with the bone block portion, which will be docked within the tibia (Fig 4, A-E). The bone block is bulletized, and the soft tissue tendon portion of the graft are shaped to pass easily within a 10-mm tunnel (Fig 4A). A 2.5-mm drill hole is created in the midportion of the bone block in the coronal plane (medial to lateral). A 2.5-mm tape suture (Dynatape, Depuy-Mitek, Johnson and Johnson, Raynham, MA) is then passed through the 2.5-mm drill hole in the bone block (Fig 4, B and C), and a separate #2 high tensile strength traction suture (Orthocord, Depuy-Mitek, Johnson and Johnson) is passed through the distal end of bone block, which will be used to assist with eventual graft passage (Fig 4, B and C). Two separate #2 high tensile strength sutures (Orthocord , Depuy-Mitek, Johnson and Johnson) are then passed into the tendinous portion of the graft in a Krackow fashion, with one placed at the end of the graft and the other at the midpoint of the graft (Fig 4, D and E). These tagging sutures improve shuttling of the graft by allowing initial traction to be pulled through the end of the graft and later pulling traction through the middle of the graft, as it is pulled further through the tunnels.

**Fig 5:**
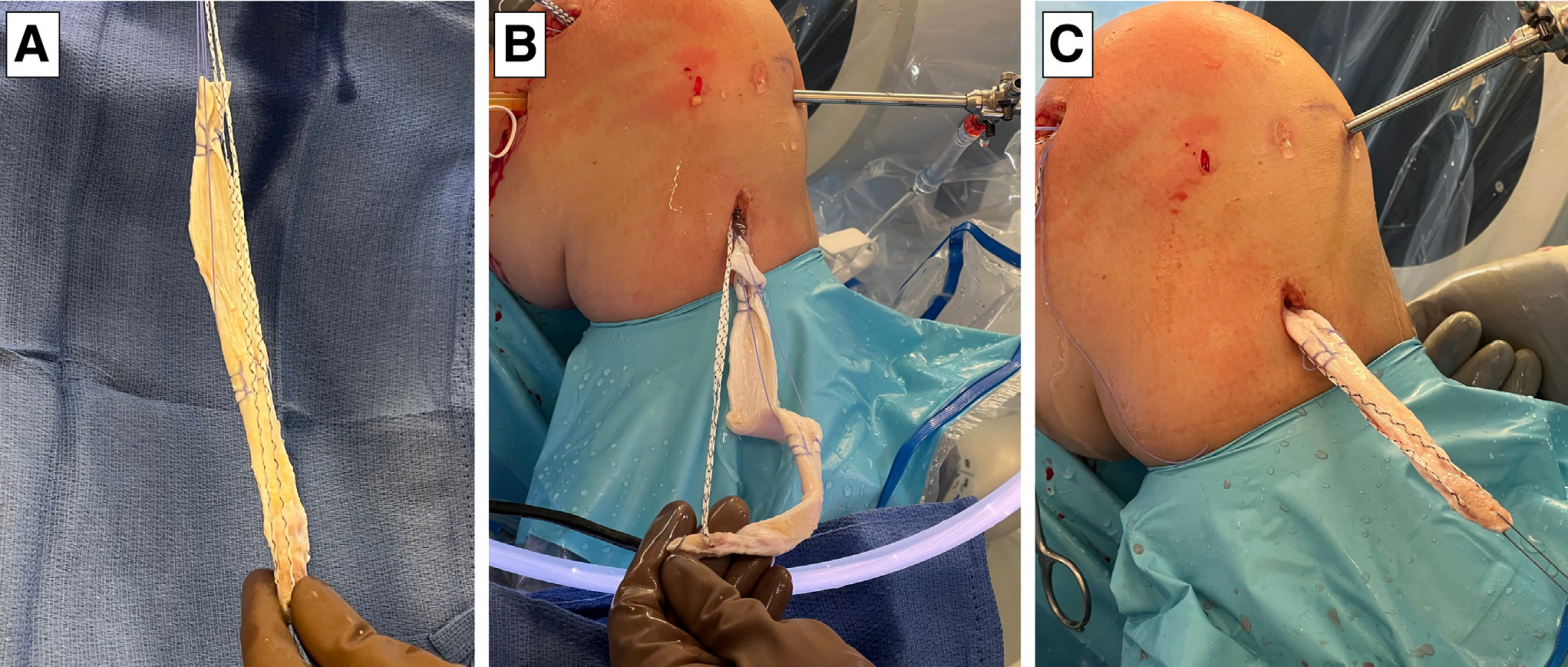
The graft is positioned for passage into the tunnels (A). In this patient’s left knee, the sutures in the tendinous portion of the graft and the suture tape in the bone block are combined at one end of the graft and then shuttled through the tunnels in a retrograde fashion, entering the joint outside-in through the tibial tunnel and retrieved out the femoral tunnel, with the use of a passing suture (B). The suture at the tip of the bone block is not shuttled with the other sutures, and it remains on the side of the tibial tunnel to assist with graft passage (C).

**Fig 6:**
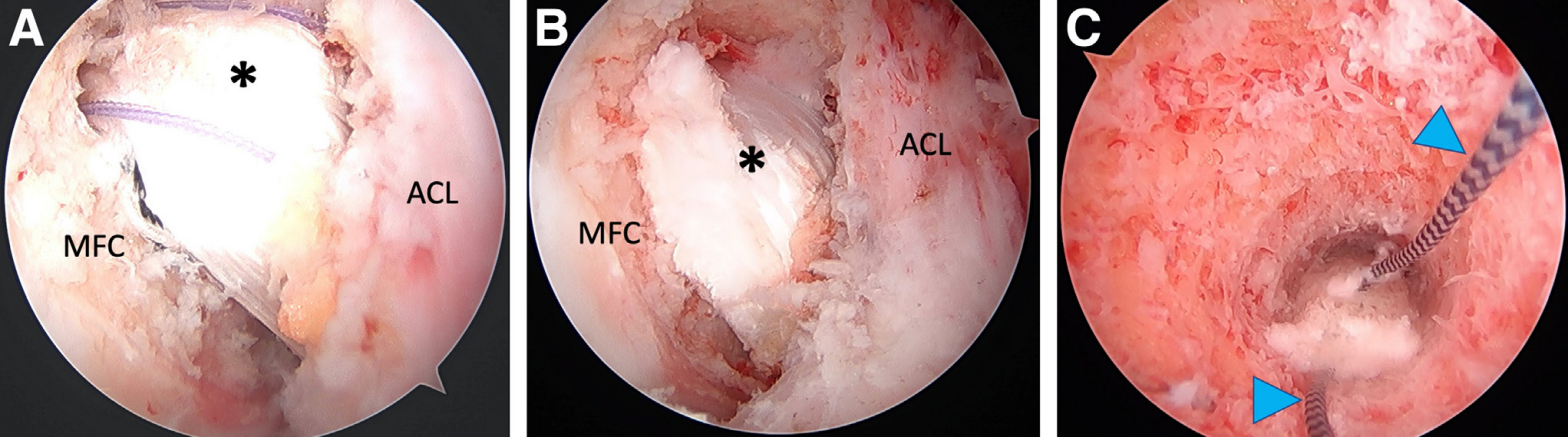
Tension is pulled on the suture limbs exiting the femoral tunnel to pass the graft (black asterisk) through the tunnels (A). The graft is fully seated (B), and the sutures placed in the midportion of the graft should no longer be visible within the tunnel. With the graft fully seated in the tunnel, the arthroscope can be placed into the tibial tunnel to confirm the bone block is at the aperture of the tibial plateau (C). The sutures placed within the soft tissue portion of the bone plug can be visualized within the tunnel (blue arrow). ACL, anterior cruciate ligament; MFC, medial femoral condyle.

**Fig 7:**
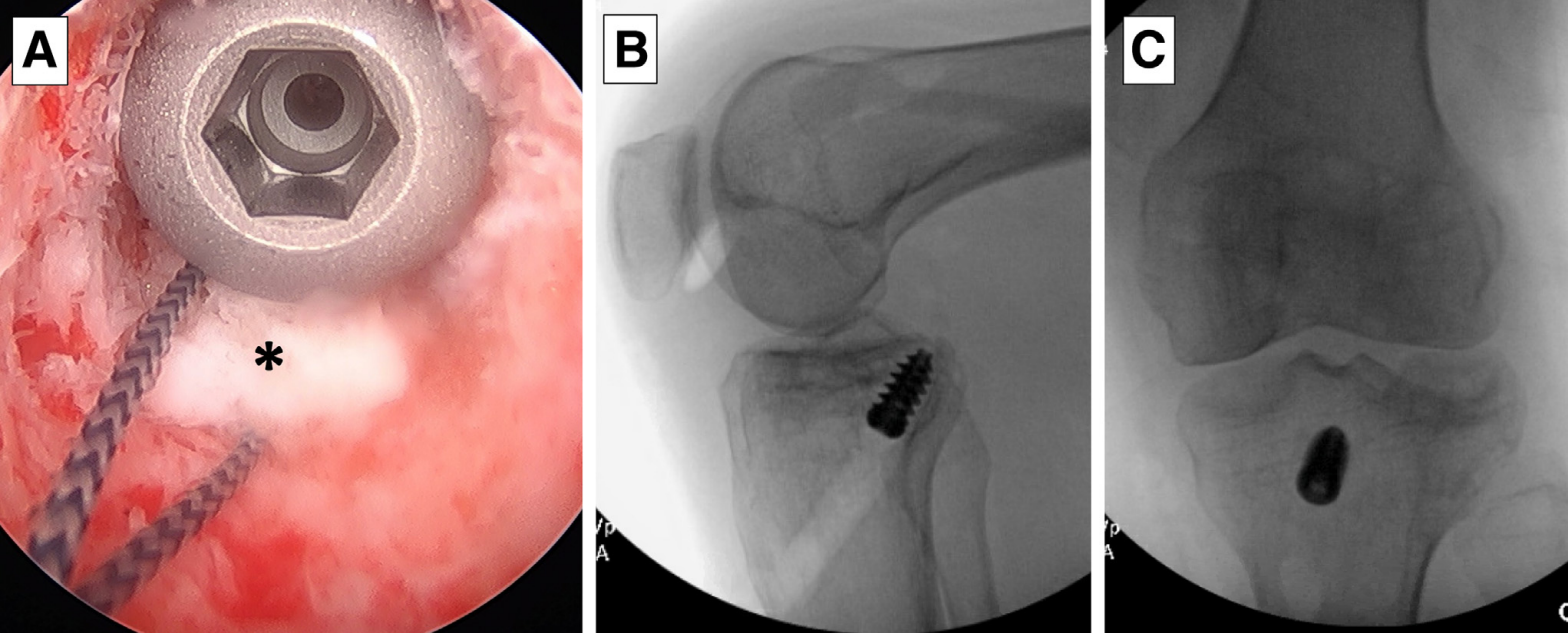
The bone plug (black asterisk) is fixed in place using a metal interference screw, seen here arthroscopically through the tibial tunnel (A) and confirmed to be in a good position on lateral and anteroposterior fluoroscopic imaging (B and C). With the bone block at the aperture of the tibial plateau, the soft tissue graft is intra-articular and avoids contact with the tibial plateau which prevents a “killer turn.”

After graft preparation, the graft will be passed in a retrograde fashion from the tibial side to the femoral side. Graft passage begins by passing a free suture retrograde into the joint from the tibial tunnel and then retrieving it from the femoral tunnel. This suture is used to shuttle both sets of tagging sutures within the soft tissue portion of the allograft and the Dynatape augment that was passed through the bone plug through the tibial tunnel into the joint, and then out the femoral tunnel (Fig 5, A-C). Traction is then pulled on the suture limbs exiting the femur until the bone plug of the graft is fully seated in the most proximal extent of the tibial tunnel, which can be confirmed arthroscopically (Fig 6, A-D). The bone plug is then fixed in place using a 9-mm metal interference screw (Fig 7A), and its position is confirmed fluoroscopically (Fig 7, B and C).

**Fig 8:**
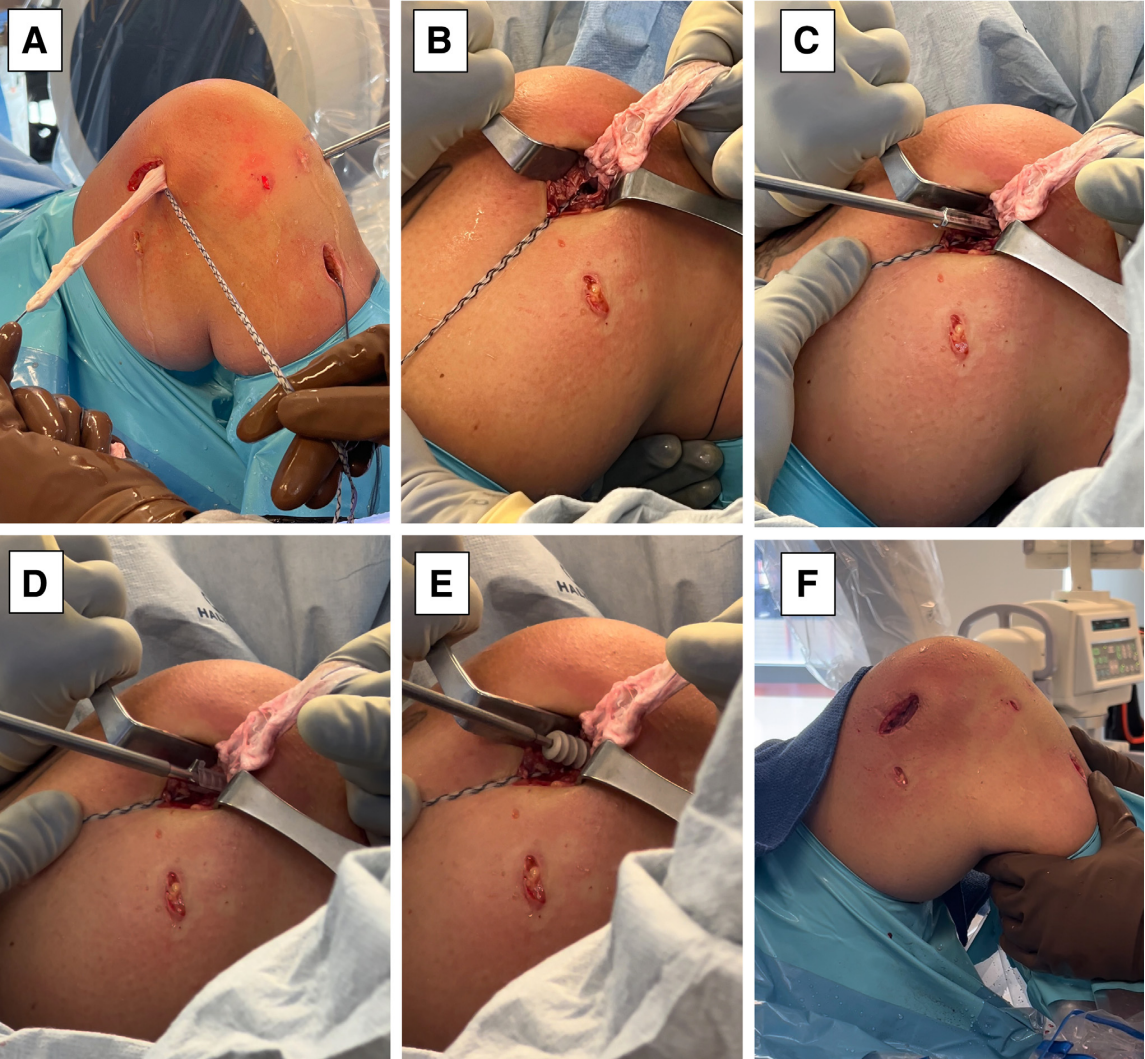
Final graft fixation in the femur is then performed in this patient’s left knee. The knee is positioned at 90° of flexion, and an anterior drawer is performed, while the graft is fixed. The allograft and tape augment are then separated from one another (A) to expose the tunnel (B). A metal dilator is then introduced into the tunnel (C), followed by insertion of an appropriately sized sheath (same size at the tunnel: 10 mm), which should be made flush with the cortex of the bone and with the allograft and tape on the opposite side of one another (D). A screw is then advanced into the center of the sheath (E), and the excess allograft and tendon are excised.

The graft is then ready for final fixation in the femur. The knee is positioned in 90° of flexion, and the anterior drawer is performed by an assistant while the graft is being fixed. Tension is applied to both the Achilles tendon allograft, and the Dynatape suture exiting the femur, in opposite directions, so that the interference screw and sheath fixation can be inserted in the center between the graft and tape augment (Fig 8, A-F). A dilator is first advanced into the tunnel, followed by placement of an appropriately sized sheath (same size as the tunnel [10 mm]), which is made flush with the femoral cortex. A screw is then advanced into the center of the sheath. It is essential to maintain tension on the graft and tape during all steps of femoral sheath and screw placement. No additional fixation or post is needed, as the sheath and screw construct creates a robust interference fixation of both the graft and the tape, which would not be possible with an interference screw alone. A posterior drawer exam is then performed to confirm an adequate reconstruction.

The excess length from the limbs of the graft and tape are cut flush with the femoral cortex. Wounds are irrigated and closed, sterile dressing are applied, a hinged knee brace is placed, and a standard postoperative rehabilitation program for PCL reconstruction ensues. Advantages and disadvantages (Table 1), as well as pearls and potential pitfalls (Table 2) of this technique, are summarized.

**Table 1:** Advantages and Disadvantages

Advantages	Disadvantages
• Minimizes killer turn with bone plug at top of tibial tunnel • No added implant cost for tape augment fixation • No graft tension mismatch as fixed through same sheath and screw	• Minor increase in cost of tape for augment • Potential stress shielding of allograft by tape • Difficult to augment both bundles with perform double-bundle PCL reconstruction

PCL, posterior cruciate ligament.

**Table 2:** Pearls and Pitfalls

Pearls	Pitfalls
• Place AM portal close to medial border of tendon to improve visualization into posterior compartment of knee • Ensure optimal placement of PM portal by placing knee at 90° of flexion, viewing from AL portal, and ensuring trajectory of spinal needle can access the posterior edge of the tibial plateau • Ensure femoral drill and reamer trajectory is posterior and deep to articular surface to prevent iatrogenic cartilage injury • Improve ease of graft passage by using an acorn femoral reamer to chamfer the posterior aspect of the tunnel • Tagging suture in the midpoint of the allograft between the bone block and tail can be used to help shuttle graft through the tunnels	• Tibial fixation of the graft and tape augmentation with an interference screw alone (i.e., without a sheath) risks wrapping of the graft and/or tape around the threads and damage to the graft and/or tape • Burying the tibial screw beneath the outer cortex risks weakening the overall fixation as tight cortical fixation is an essential component of the sheath-screw construct • Unequal tensioning of the graft and tape during final tibial fixation risks a mismatch in the length-tension relationship of the graft and tape, which limits the benefit of this construct

AM, anteromedial; PM, posteromedial.

## Discussion

A variety of techniques for posterior cruciate ligament reconstruction exist, including single- and double-bundle reconstruction, as well as inlay and onlay type reconstuctions.^2^^,^^3^ Although many techniques have been described, residual laxity remains a consistent problem in patients undergoing PCL reconstruction.^8^ In order to counteract PCL graft elongation and the resulting laxity, an “internal brace” or suture/tape augmentation has been added to many reconstructive procedures. In biomechanical studies, tape augmentation has been shown to diminish graft elongation and improve ultimate strength.^9^ Despite the promising biomechanical data, long-term clinical data are lacking, and there remains concern among surgeons that improperly tensioned suture or tape augments could lead to stress shielding of the incorporating graft. Our technique was developed to allow for simultaneous tensioning of the PCL graft and the internal brace in order to minimize the risk of stress shielding on the incorporating graft.

In this technique, the 2.5-mm tape suture is secured distally by passing it through a drill hole in the Achilles graft bone block. This allows the suture tape to be linked directly to the graft and avoids the need for independent tibial fixation of the augment. After the graft and augment are passed into the femoral tunnel, both are tensioned and secured simultaneously with a sheath and screw construct. The use of the sheath and screw allows for robust interference fixation and fully eliminates the need for independent tensioning and fixation of the tape augment. By simultaneously tensioning the graft and internal brace, this technique aims to minimize the risk of stress shielding and allows the graft be incorporated under physiological loads, while decreasing the risk for graft elongation. In addition, it reduces operative time and cost by eliminating the need for placement of independent hardware for fixation of the tape.

Another technical consideration when performing PCL reconstruction is managing the so-called “killer turn” at the proximal aperture of the tibial tunnel.^10^ The “killer turn” causes difficulty with graft shuttling and has also been implicated in graft attenuation.^11^ Our technique uses specific steps to mitigate both of these challenges. By placing the bone plug within the tibia and passing the graft in a retrograde fashion, we are able to avoid the difficulty of shuttling a bone-block around the killer turn. Second, by bringing the bone block to the proximal aperture of the tunnel, we minimize draping the soft tissue portion of the graft over the anterior edge of the tibial tunnel. This is done to decrease point loading of the graft at the killer turn and minimize the risk of graft elongation.

In this article, we present a surgical technique for single-bundle PCL reconstruction with postless tape augmentation. This technique provides the additional mechanical stability of tape augmentation without the concerns associated with independent tensioning from the graft. In addition, it eliminates the additional cost and steps associated with post-type fixation using additional tape. Finally, this technique makes several modifications to previously described PCL reconstruction techniques in order to minimize the complications associated with the “killer turn”.

## Supplementary Data

ICMJE author disclosure forms

Video 1 This technique demonstrates a case using postless tape augmentation during posterior cruciate ligament (PCL) reconstruction. The tibial and femoral tunnels are first created with the assistance of an accessory posteromedial portal and drilling under direct arthroscopic and fluoroscopic visualization. An Achilles tendon allograft with a bone plug is used as the allograft. The bone plug and soft tissue are shaped to fit within 10-mm tunnels. A 2.5-mm suture tape (Dynatape, Depuy-Mitek, Johnson and Johnson, Raynham, MA) is then passed through a 2.5-mm drill hole in the bone block, which will be used as the postless augment for the graft. High-tensile strength suture (#2 Orthocord, Depuy-Mitek, Johnson and Johnson) is then passed through the end of the bone block and two additional high-tensile strength sutures (#2 Orthocord, Depuy-Mitek, Johnson and Johnson) are sutured into the soft tissue portion of the graft, in a Krackow suture pattern, to assist with graft passage. The graft is then passed through the tunnels in a retrograde fashion with the assistance of a shuttling suture. Once the graft has been passed out the femur, and the bone plug is fully seated in the most proximal extent of the tibial tunnel, it is secured in place using a metal interference screw, which is confirmed fluoroscopically. Final fixation of the graft is then performed with the knee placed at 90° of flexion, application of an anterior drawer, and insertion of a screw and sheath into the tunnel. It is essential to maintain tension on the allograft and tape during all steps of tibial screw and sheath placement. No additional fixation is required in the femur, as both the allograft and tape are well secured with the sheath and screw.

## References

[1] Bedi A., Musahl V., Cowan J. (2016). Management of posterior cruciate ligament injuries: An Evidence-based review. J Am Acad Orthop Surg.

[2] Kim S., Kim T., Jo S., Kung Y. (2009). Comparison of the clinical results of three posterior cruciate ligament reconstruction techniques. J Bone Joint Surg Am.

[3] Yoon K., Kim E., Kwon Y., Kim S. (2019). Minimum 10-year results of single- versus double-bundle posterior cruciate ligament reconstruction: Clinical, radiologic, and survivorship outcomes. Am J Sports Med.

[4] Shin Y., Kim H., Lee D. (2017). No clinically important difference in knee scores or instability between transtibial and inlay techniques for PCL reconstruction: A systematic review. Clin Orthop Relat Res.

[5] Krott N., Wengle L., Whelan D., Wild M., Betsch M. (2022). Single and double bundle posterior cruciate ligament reconstruction yield comparable clinical and functional outcomes: A systematic review and meta-analysis. Knee Surg Sports Traumatol Arthrosc.

[6] MacGillivray J., Stein B., Park M., Allen A., Wickiewicz T., Warren R. (2006). Comparison of tibial inlay versus transtibial techniques for isolated posterior cruciate ligament reconstruction: Minimum 2-year follow-up. Arthroscopy.

[7] Levy B., Piepenbrink M., Stuart M., Wijdicks C. (2021). Posterior cruciate ligament reconstruction with independent suture tape reinforcement: An in vitro biomechanical full construct study. Orthop J Sports Med.

[8] MacGillivray J.D., Stein B.E., Park M., Allen A.A., Wickiewicz T.L., Warren R.F. (2006). Comparison of tibial inlay versus transtibial techniques for isolated posterior cruciate ligament reconstruction: minimum 2-year follow-up. Arthroscopy.

[9] Levy B.A., Piepenbrink M., Stuart M.J., Wijdicks C.A. (2021). Posterior cruciate ligament reconstruction with independent suture tape reinforcement: An in vitro biomechanical full construct study. Orthop J Sports Med.

[10] Vasdev A., Rajgopal A., Gupta H., Dahiya V., Tyagi V.C. (2016). Arthroscopic all-inside posterior cruciate ligament reconstruction: Overcoming the "killer turn". Arthrosc Tech.

[11] Weimann A., Wolfert A., Zantop T., Eggers A.K., Raschke M., Petersen W. (2007). Reducing the "killer turn" in posterior cruciate ligament reconstruction by fixation level and smoothing the tibial aperture. Arthroscopy.

